# Theory-Based Predictors of Mindfulness Meditation Mobile App Usage: A Survey and Cohort Study

**DOI:** 10.2196/10794

**Published:** 2019-03-22

**Authors:** AliceAnn Crandall, Aaron Cheung, Ashley Young, Audrey P Hooper

**Affiliations:** 1 Brigham Young University Department of Public Health Provo, UT United States

**Keywords:** executive function, intention, mindfulness, mobile apps, social theory

## Abstract

**Background:**

Mindfulness meditation has become increasingly popular over the last few years, due in part to the increase in mobile apps incorporating the practice. Although studies have demonstrated the potential of mindfulness meditation to positively impact health, little has been uncovered about what predicts engagement in mindfulness meditation. Understanding the predictors of mindfulness meditation may help practitioners and phone app developers improve intervention strategies and app experience.

**Objective:**

The purpose of this study was to use the Theory of Planned Behavior and Temporal Self-Regulation Theory to determine factors predicting mindfulness meditation mobile app use.

**Methods:**

The sample consisted of 85 undergraduate students with no prior mindfulness meditation experience. During their first laboratory visit, participants completed tasks to measure their executive functioning and a survey to measure Theory of Planned Behavior constructs about mindfulness meditation. Over the following 2 weeks, participants logged the days and minutes that they practiced mindfulness meditation using a phone app. Hierarchical regression modeling was used to analyze the data.

**Results:**

After controlling for demographic factors, participant subjective norms (beta=14.51, *P*=.001) and intentions (beta=36.12, *P*=.001) were predictive of the number of minutes practicing mindfulness. Participant executive functioning did not predict mindfulness meditation practice, nor did it moderate the link between intentions and mindfulness meditation practice. Participant attitudes (beta=0.44, *P*<.001) and perceived control (beta=0.42, *P*=.002) were positively associated with intentions to practice mindfulness.

**Conclusions:**

These results suggest that among college student populations, the Theory of Planned Behavior may be useful in predicting the use of mindfulness meditation phone apps. However, participant executive functioning was not a predictor or moderator of mindfulness practice, and Temporal Self-Regulation Theory may be less useful for explaining mindfulness meditation behaviors using phone apps over a short period of time among college students. The results have implications for public health professionals, suggesting that a focus on subjective norms and intentions may promote mindfulness meditation practice using phone apps.

## Introduction

Mindfulness is the practice of being aware of the present moment [1]. In recent years, mindfulness meditation has become especially popular in the Western world and is increasingly used as a form of medical and psychological therapy [2] due to its perceived benefits to mental and physical health. Although people can be mindful without meditating, mindfulness meditation guides can help people acquire the skill [3]. There are numerous ways to learn how to practice mindfulness, including mindfulness meditation retreats, small group classes, and audio-based training. More recently, several mindfulness meditation phone apps (both paid and free versions) have been introduced on the market [4].

Prior studies have demonstrated several benefits of being mindful. The benefits to mental health are the most widely studied. For example, studies have found associations between mindfulness meditation and increased behavioral regulation and executive functioning [5,6], and decreased psychological symptoms and emotional reactivity [7]. Mindfulness meditation has also been associated with improved academic performance [8], improved well-being [7,9], and better quality of life [10,11]. Even more promising is that these changes may be sustained over time [11]. Benefits also extend to physical health [5], including that it may help to manage and treat chronic diseases [12-15] and improve immune function [16]. For example, a study conducted in a work environment of 41 employees examined the effectiveness of mindfulness meditation on immune function before receiving the flu vaccination [16]. Participants who practiced mindfulness meditation had increased levels of antibody titers to the influenza virus compared to those who did not practice mindfulness.

### Mindfulness Phone Apps

Few clinical trials have been conducted assessing the effectiveness of mindfulness meditation mobile phone apps. However, the extant literature suggests that mindfulness mobile apps show promise in their ability to effectively increase mindfulness in university students, even compared to other tactics such as audio-based training [17]. Another study found that in an adult population a 5-week self-help intervention guided by a mindfulness app significantly improved quality of life and these gains were maintained for at least 3 months, demonstrating the possibility for mindfulness apps to achieve durable positive effects [11].

The potential of mindfulness phone apps remains largely unexplored [18]. A review of several mindfulness apps on the market found that few apps consistently performed well across areas of user engagement, functionality, visual esthetics, information quality, and subjective quality [4]. There is also limited research addressing what exactly predicts mindfulness app use and what will help make these apps a reliable tool for various populations moving forward.

Given the many perceived benefits of mindfulness meditation, it is important to know what encourages or predicts engagement in mindfulness meditation in the general population. Such information could help to inform intervention efforts. However, little is currently known about predictors of mindfulness meditation, particularly among those with limited exposure to the practice. Understanding predictors of mindfulness meditation and mindfulness phone app usage would aid in developing more effective health promotion interventions aimed at increasing mindfulness. Two theoretical frameworks, the Theory of Planned Behavior and Temporal Self-Regulation Theory, may be useful in predicting mindfulness meditation engagement.

### Theoretical Framework

The purpose of this study was to examine the predictors of engaging in mindfulness meditation using phone apps among individuals with no prior exposure to mindfulness. The Theory of Planned Behavior [19] and Temporal Self-Regulation Theory [20] provided the theoretical framework for this study. The Theory of Planned Behavior is widely used across disciplines and in particular for understanding health behaviors [21,22]. Based on the Theory of Planned Behavior, intentions drive behavior. Intentions are formed from attitudes and beliefs about the behavior, perceptions of social norms regarding the behavior (ie, subjective norms), and perceived behavioral control. More recently, meta-analyses of studies that have used the Theory of Planned Behavior have demonstrated that intentions have a small-to-medium effect on behavior [21,22] and that the age of the participant and type of behavior being studied may affect the intention-behavior link [22]. Considering these factors, researchers increasingly are considering biological factors, including cognitive factors, which may influence the relationship between intentions and behavior [23].

Temporal Self-Regulation Theory may help to explain the modest association between intention and behavior. This theory posits that an individual’s executive functioning capacity may directly predict behavior or moderate the association between intention and behavior [20]. Executive functions are goal-directed capacities including working memory, inhibitory control, and cognitive shifting [24]. These capacities allow the brain to exercise top-down control of behavior (eg, executive functioning may directly predict the health behavior). Furthermore, executive functions may also enable individuals to more easily act on their intentions due to their increased ability to inhibit prepotent responses in favor of a longer-term goal and to better focus their attention and motivation on achieving these goals.

Prior studies have used Temporal Self-Regulation Theory as the theoretical framework to predict physical health behaviors with mixed results. Hall and colleagues [25] found that executive functions added unique variance to models testing the relationship between intention and physical activity and between intention and dietary behaviors among undergraduate students. For both physical activity and dietary behaviors, they also found that the association between intentions and behaviors was stronger among participants with higher executive functioning [25]. Conversely, in an online sample, Evans and colleagues [26] examined whether self-control, an aspect of inhibitory control, predicted fruit and vegetable and unhealthy snack consumption. They found no relationship between self-control and these dietary behaviors, nor did self-control moderate the intention-to-behavior link.

Although the Theory of Planned Behavior and Temporal Self-Regulation Theory have not previously been linked with mindfulness meditation app usage, prior research has indicated a relationship between the Theory of Planned Behavior and other technology use. For example, in a study of university students, it was found that attitudes and subjective norms (but not perceived behavioral control) significantly predicted intentions to engage in high-level social networking websites, and intentions predicted actual behavior [27]. A study of junior and senior university students found that intentions to use information systems were predicted by attitudes and perceived behavioral control. Subjective norms did not predict intentions to use information systems [28].

### Aims and Hypotheses

To our knowledge, no studies have examined theoretical constructs for why people practice mindfulness meditation. Furthermore, although Temporal Self-Regulation Theory has been associated with some physical health behaviors, it has not been tested on mental health behaviors such as mindfulness meditation. In this study, we hypothesized that (1) in accordance with the Theory of Planned Behavior, participant attitudes about mindfulness meditation practice, subjective norms, perceived behavioral control, and especially their intentions to practice mindfulness would predict the number of days and minutes of mindfulness meditation practice using a phone app, and (2) using Temporal Self-Regulation Theory, we hypothesized that individual executive functioning capacity would moderate the link between intentions and behaviors, such that the association between intentions and behaviors would be stronger among those with stronger executive functioning ability. We included measures of both days and minutes of mindfulness meditation app use to examine consistency of practicing mindfulness meditation (number of days) and the length of time spent practicing (number of minutes).

## Methods

### Recruitment and Procedures

The sample consisted of 85 undergraduate students at a university in the Intermountain West. Participants were recruited via in-class announcements and fliers posted around campus. Eligibility requirements were (1) participants must be currently enrolled as undergraduate students, and (2) participants must have had no prior or minimal exposure (less than 2 hours) to mindfulness meditation.

On enrolling in the study, two laboratory visits were scheduled with the participant. At the first laboratory visit, participants signed their consent to participate in the study. They were also introduced to mindfulness and mindfulness meditation using the Smiling Mind App. The Smiling Mind App was recommended to participants because it was free, compatible with Android and Apple products, and had been reviewed as one of the top mindfulness phone apps available [4,29]. Additionally, this app provides guided meditation for a variety of demographics and situations (eg, by age group, for work and sports, mindfulness for managing stress or improving relationships) and has flexibility in the length of each mindfulness meditation session (eg, from just over 1 minute to 30-60 minutes) [30]. Research personnel helped the participant download the app on their phone during the laboratory visit. Participants were told that they could use other mindfulness meditation apps should they choose and could also access these same programs online if they did not want to download the app on their phone. Participants then completed executive functioning and physiological tasks followed by a brief online survey on Qualtrics. At the end of the laboratory visit, research personnel instructed participants on how to complete a mindfulness meditation log. They were informed that if they filled out the mindfulness meditation log and brought it back to the next laboratory visit that they would be entered into a drawing for one of two US$100 e-gift cards. To be eligible for the draw, participants did not have to practice mindfulness, but they did have to enter the number of minutes that they practiced mindfulness each day (0 minutes for no mindfulness practice) during the 2 weeks between laboratory visits. Participants received a reminder text, email, or phone call (depending on their preference) 1 week after the first laboratory visit to remind them of the study and to fill out their log. Approximately 24 hours before the second laboratory visit, participants received a reminder text, email, or phone call. At the second laboratory visit, participants submitted their completed mindfulness meditation log, participated in the executive functioning tasks, and completed a follow-up survey. This study focuses on the tasks and survey responses from the first laboratory visit and the number of days and minutes that participants practiced mindfulness during the two weeks of the study. The study was approved by the Brigham Young University Institutional Review Board, Provo, Utah.

The sample size of 85 was selected based on a power analysis conducted in Stata 14 using the powerreg command, estimating a power of 0.80 and assuming a 10% dropout rate and a change in *R*^2^ of .04 (based on a study using the Theory of Planned Behavior that examined health behaviors) [25]. We did not experience the expected 10% dropout and thus had higher power than 0.80.

### Measures

#### Mindfulness Meditation

Mindfulness meditation was measured by participant self-report using their mindfulness meditation log. We measured the number of days that students practiced mindfulness meditation between laboratory visits and the total number of minutes of mindfulness practiced.

#### Theory of Planned Behavior Constructs

In the survey that participants completed during the first laboratory visit, participants reported on their attitudes about mindfulness meditation, subjective norms about mindfulness, perceived behavioral control to practice mindfulness, and their intentions to practice mindfulness. The Theory of Planned Behavior questions were created using methodology developed by Icek Ajzen [31] (see Table 1 for a complete listing of items).

**Table 1:** Theory of Planned Behavior items

Item		Response option range
**Intentions**		
	1. I intend to practice mindfulness each day in the forthcoming 2 weeks.	Extremely unlikely to extremely likely
	2. In the forthcoming 2 weeks, how often do you plan to practice mindfulness for at least 5 minutes?	Never to every day
	3. I will try to practice mindfulness for at least 5 minutes each day in the forthcoming 2 weeks.	False to definitely true
	4. I plan to practice mindfulness for at least 5 minutes each day in the forthcoming 2 weeks.	Strongly disagree to strongly agree
**Attitudes**		
	5. For me, to practice mindfulness meditation each day in the forthcoming 2 weeks is:	Harmful to beneficial
	6. For me, to practice mindfulness meditation each day in the forthcoming 2 weeks is:	Unpleasant to pleasant
	7. For me, to practice mindfulness meditation each day in the forthcoming 2 weeks is:	Bad to good
	8. For me, to practice mindfulness meditation each day in the forthcoming 2 weeks is:	Worthless to worthwhile
	9. For me, to practice mindfulness meditation each day in the forthcoming 2 weeks is:^a^	Unenjoyable to enjoyable
	10. For me, to practice mindfulness meditation each day in the forthcoming 2 weeks is:	A waste of time to an important use of my time
**Subjective norms**		
	11. Most people who are important to me think that I should practice mindfulness meditation.^a^	Strongly disagree to agree
	12. The people in my life whose opinions I value would approve of me practicing mindfulness meditation.^a^	Strongly disagree to agree
	13. Most people who are important to me practice mindfulness meditation often.	Completely false to completely true
	14. The people in my life whose opinions I value, practice mindfulness meditation often.	Completely false to completely true
**Perceived behavioral control**		
	15. For me, to practice mindfulness meditation daily would be	Impossible to possible
	16. If I wanted to, I could practice mindfulness meditation daily.	Definitely false to definitely true
	17. How much control do you believe you have over practicing mindfulness meditation daily?	No control to complete control
	18. It is mostly up to me whether or not I practice mindfulness meditation daily.	Strongly disagree to agree

^a^Item dropped during exploratory factor analysis.

Four items were used to measure intentions to practice mindfulness. For three questions, answer options were on a four-point scale; one question was on a seven-point scale (“In the forthcoming 2 weeks, how often do you plan to practice mindfulness for at least 5 minutes?” with answer options ranging from “never” to “every day”) *.* Due to low response counts for practicing mindfulness on fewer than half of the days, and to be consistent with the other intentions questions, response options were combined for a four-point scale. There were six items in the attitudes subscale. Each item was measured on a five-point scale, with higher scores indicating more positive attitudes about engaging in mindfulness meditation. Due to low counts for negative or neutral perceptions about practicing mindfulness, we combined negative and neutral responses so that each item was analyzed on a three-point scale (0=negative or neutral attitudes; 1=somewhat positive attitudes; 2=strong agreement or strongly positive attitudes about practicing mindfulness). We asked four questions relating to subjective norms pertaining to practicing mindfulness meditation. Response options ranged from 1=strongly disagree/completely false to 5=agree/completely true. Perceived behavioral control about practicing mindfulness was measured through four items on a five-point scale.

#### Executive Functioning

Executive functioning was measured using three tasks from the NIH Toolbox (NIH-TB) [32]. We used the NIH-TB Dimensional Change Card Sort Test to measure cognitive shifting and the NIH-TB Flanker Inhibitory Control and Attention Test to measure inhibitory control and attention. We used the age-standardized scores and averaged participant scores across the two tasks. To assess working memory, we used the age-standardized score of the NIH-TB List Sorting Working Memory Test.

### Demographic Controls

We controlled for participant gender (0=male; 1=female), age, employment status (0=not employed; 1=employed), and performance on the NIH-TB Picture Vocabulary Test [32] (age-standardized scores) to control for participant fluid intelligence (considered best practice when examining executive functioning [33]).

### Statistical Analysis

We conducted exploratory factor analysis for one-, two-, three-, and four-factor models to examine whether the data fit the factor structure of the Theory of Planned Behavior constructs using Mplus Version 7. We assessed the factor loadings and two model fit indexes: the comparative fit index (CFI; >0.90 indicates adequate fit) and the root mean square error of approximation (RMSEA; <0.08 indicates adequate fit). Items with factor loadings <0.40 or with a cross-loading >0.30 on a second factor were dropped [34].

Descriptive statistics and hierarchical regression analyses were carried out using Stata Version 14. Using Stata’s nestreg command, we used five blocks of data in a hierarchical linear regression process to test our hypotheses. In the first block, demographic factors were included. The second block included Theory of Planned Behavior constructs (attitudes, subjective norms, and perceived behavioral control). Participant intentions to practice mindfulness were added in the third block, and executive function (as a measure relating to Temporal Self-Regulation Theory) was added in the fourth block. The fifth block included interaction terms to examine whether executive functioning and working memory moderated the intention-behavior link.

## Results

### Sample Description

Approximately half of the participants were female (45 of 85 participants; 53%), 60 of 85 participants (80%) identified themselves as white or Caucasian, 20 (24%) were married, and 47 (56%) of participant’s mothers had a bachelor’s degree or higher. Table 2 contains the means and standard deviations of study variables.

### Factor Analysis

Exploratory factor analysis demonstrated that a four-factor model fit the data best for the Theory of Planned Behavior items (RMSEA=0.040, CFI=0.997). Two items from the subjective norms subscale and one item from the attitudes subscale were below the minimum cutoff or had high cross-loadings on a second factor and were dropped. The remaining loadings were all above the minimum cutoff, with loadings >0.75 for factor 1 (intentions), >0.81 for factor 2 (attitudes), >0.75 for factor 3 (perceived behavioral control), and >0.91 for factor 4 (subjective norms). The Cronbach alpha for all subscales ranged from adequate to high (intentions: Cronbach alpha=.86; attitudes: Cronbach alpha=.90; subjective norms: Cronbach alpha=.70; perceived behavioral control: Cronbach alpha=.68).

Items were summed and averaged for each of the four constructs. Higher scores in each of the four constructs indicated greater intent to practice mindfulness, more positive attitudes about mindfulness meditation, higher perceived behavioral control to engage in mindfulness meditation, and stronger subjective norms to practice mindfulness, respectively.

### Descriptive Results: Theory of Planned Behavior, Mindfulness, and Executive Functioning

On average, participants practiced mindfulness on 9.3 (SD 3.6) days and for 64.3 (SD 44.0) minutes over the 14 days, with a range from 0 minutes (two participants) to 210 minutes. In general, participants had very positive attitudes toward mindfulness (mean 1.54, SD 0.53; scale 0-2 points), but report of subjective norms was low (mean 1.03, SD 1.01; scale 0-4 points). Participants on average felt that they had very high control over practicing mindfulness (mean 3.73, SD 0.38; scale 0-4 points). Intentions to practice mindfulness were generally high, with an average score of 2.83 (SD 0.53; scale 0-4 points). The mean executive functioning score was 100.7 (SD 13.5) with 15 of 85 (18%) participants scoring below one standard deviation of the population age-standardized mean and 12 of 85 (14%) participants scoring one standard deviation or above the population mean. The mean score on the working memory task was 105.6 (SD 13.0) with 4 of 85 (5%) participants scoring below one standard deviation of the population age-standardized mean and 24 of 85 (28%) scoring one standard deviation or more above the population mean.

### Correlations

Intentions to practice mindfulness were correlated with increased number of days (*r*=.43, *P*<.001) and minutes (*r*=.37, *P*<.001) practicing mindfulness. Neither executive functioning nor working memory were correlated with days (executive functioning: *r*=–.16, *P*=.15; working memory: *r*=–.04, *P*=.72) or minutes (executive functioning: *r*=–.19, *P*=.08; working memory: *r*=–.04, *P*=.70) of mindfulness meditation (see Table 3).

**Table 2:** Descriptive statistics of demographic and key study variables (N=85).

Study variable	Mean (SD)
Proportion female	0.53 (0.50)
Age	21.81 (2.60)
Proportion employed	0.74 (0.44)
NIH Toolbox Picture Vocabulary Test	117.39 (11.86)
Attitudes^a^	1.54 (0.53)
Subjective norms^b^	1.03 (1.01)
Perceived behavioral control^b^	3.73 (0.38)
Intentions^b^	2.83 (0.53)
Executive functioning	100.65 (13.49)
Working memory	105.60 (13.00)
Days of mindfulness	9.29 (3.56)
Minutes of mindfulness	64.31 (43.95)

^a^Attitude items were on a scale of 0-3 points.

^b^Subjective norms, perceived behavioral control, and intentions were on a scale of 0-4 points.

**Table 3:** Correlations (*r*) of demographic and key study variables (N=85).

Variable name	1	2	3	4	5	6	7	8	9	10	11	12
1. Gender^a^	1.00											
2. Age	–0.29^b^	1.00										
3. Employed^c^	–0.18	0.37^b^	1.00									
4. NIH Toolbox Picture Vocabulary Test	–0.08	–0.22^b^	–0.10	1.00								
5. Attitudes^d^	–0.03	0.14	0.14	–0.06	1.00							
6. Subjective norms^e^	0.02	0.23^b^	0.02	–0.07	0.23^b^	1.00						
7. Perceived behavioral control^e^	0.08	–0.17	0.03	–0.03	0.28^b^	0.05	1.00					
8. Intentions^e^	–0.06	0.06	0.12	–0.09	0.54^b^	0.15	0.43^b^	1.00				
9. Executive functioning	–0.10	0.10	0.08	–0.11	–0.10	0.05	0.07	–0.14	1.00			
10. Working memory	0.10	–0.18	0.09	0.38^b^	–0.19	0.06	0.20	–0.08	0.17	1.00		
11. # Days mindfulness	0.01	0.02	0.03	0.03	0.17	0.23^b^	0.10	0.44^b^	–0.16	–0.04	1.00	
12. # Minutes mindfulness	0.09	0.05	–0.08	0.02	0.22^b^	0.38^b^	0.01	0.37^b^	–0.19	–0.04	0.68^b^	1.00

^a^0=male, 1=female.

*These pairwise correlations are significant (*P*<.05).

^b^0=not employed, 1=employed.

^c^Attitude items were on a scale of 0-3 points.

^e^Subjective norms, perceived behavioral control, and intentions were on a scale of 0-4 points.

### Hierarchical Regressions

In the hierarchical regressions (see Tables 4 and 5), participant demographic factors did not contribute significantly to the models (*F*_4,80_*=* 0.05, *R*^2^=.003, *P*=.99). For number of days practicing mindfulness (Table 4), participant attitudes, subjective norms, and perceived behavioral control did not significantly explain the variance in the model (*F*_3,77_=2.00, Δ *R*^2^=.07, *P*=.12), but the addition of behavioral intentions (beta=3.52, *P*<.001) in step 3 was significant (*F*_1,76_*=* 17.29 *,* Δ *R*^2^=.17, *P*<.001). The addition of executive functioning and working memory in step 4 (*F*_2,74_*=* 0.46, Δ *R*^2^=.01, *P*=.63) and the inclusion of interaction terms in step 5 (*F*_2,72_ =0.08, Δ *R*^2^=.002, *P*=.92) did not significantly contribute to the model.

For number of minutes practicing mindfulness (Table 5), demographic variables did not contribute significantly to the model (*F*_4,80_=0.57, *R*^2^=.03, *P*=.68). The addition of attitudes, subjective norms, and perceived behavioral control to the model significantly improved the variance explained by the model (*F*_3,77_=5.13, Δ *R*^2^=.16, *P*=.003), with subjective norms significantly associated with more minutes practicing mindfulness (beta=15.19, *P*=.002). The addition of intentions (beta=36.12, *P*=.001) in step 3 also significantly explained the variance of the model (*F*_1,76_*=* 13.07, Δ *R*^2^=0.12, *P*<.001) and subjective norms also remained significant (beta=14.51, *P*=.001). As with the model illustrating the number of days, executive functioning and working memory did not contribute significantly to the model for number of minutes practicing mindfulness (*F*_2,74_=0.64, Δ *R*^2^=.01, *P*=.53), nor did executive functioning and working memory significantly moderate the intention-to-behavior link (*F*_2,72_=0.14, Δ *R*^2^=.003, *P*=.87).

### Sensitivity Analysis

Adding interaction terms to a model may result in multicollinearity; therefore, we next *z*-score standardized the variables to examine if results changed [35]. Results were substantively the same as the models reported in Tables 3 and 4.

**Table 4:** Hierarchical regression analyses: Theory of Planned Behavior constructs and executive function as predictors of number of days using a mindfulness meditation phone app (N=85).

Variable	Step 1		Step 2		Step 3		Step 4		Step 5	
	Beta	*P* value	Beta	*P* value	Beta	*P* value	Beta	*P* value	Beta	*P* value
Gender	0.19	.82	0.04	.96	0.32	.67	0.30	.70	0.38	.64
Age	0.03	.85	–0.05	.80	–0.04	.81	–0.04	.81	–0.06	.76
Employed	0.21	.83	0.20	.84	0.04	.96	0.18	.84	0.24	.80
NIH Toolbox Picture Vocabulary Test	0.01	.74	0.01	.69	0.02	.46	0.03	.43	0.03	.45
Attitudes			0.70	.38	–0.83	.31	–0.97	.26	–0.93	.29
Subjective norms			0.76	.07	0.70	.07	0.75	.05	0.76	.05
Perceived behavioral control			0.52	.64	–0.96	.37	–0.67	.55	–0.76	.55
Intentions					3.52	<.001	3.37	<.001	3.29	.65
Executive functioning							–0.02	.47	0.03	.82
Working memory							–0.02	.64	–0.07	.71
Intentions × executive functioning									–0.02	.71
Intentions × working memory									0.02	.77

**Table 5:** Hierarchical regression analyses: Theory of Planned Behavior constructs and executive function as predictors of number of minutes using a mindfulness meditation phone app (N=85).

Variable	Step 1		Step 2		Step 3		Step 4		Step 5	
	Beta	*P* value	Beta	*P* value	Beta	*P* value	Beta	*P* value	Beta	*P* value
Gender	9.92	.34	7.35	.44	10.24	.25	9.25	.31	10.08	.29
Age	2.35	.27	0.20	.93	0.26	.89	0.36	.86	0.02	.99
Employed	–10.89	.36	–9.61	.39	–11.22	.29	–10.34	.34	–9.62	.39
NIH Toolbox Picture Vocabulary Test	0.20	.64	0.20	.61	0.30	.41	0.27	.52	0.28	.50
Attitudes			14.58	.12	–1.16	.91	–2.04	.84	–1.58	.88
Subjective norms			15.19	.002	14.51	.001	14.90	.001	15.07	.002
Perceived behavioral control			–6.77	.59	–21.98	.08	–19.10	.15	–22.55	.13
Intentions					36.12	.001	33.97	.001	69.52	.42
Executive Functioning							–0.37	.28	0.43	.81
Working memory							–0.03	.94	0.11	.96
Intentions × executive functioning									–0.29	.65
Intentions × working memory									–0.05	.95

Given the importance of intentions to predicting days and minutes of mindfulness meditation practice, we conducted a hierarchical analysis with intentions at the outcome to examine the key predictors of intention to practice mindfulness. After entering participant demographic factors in step 1 (*F*_4,80_=0.51, Δ *R*^2^=.02, *P*=.73), in step 2 we found that participant attitudes (beta=0.44, *P*<.001) and perceived behavioral control (beta=0.42, *P*=.002) were both positively associated with their intentions. Subjective norms were not associated with intentions (beta=0.02, *P*=.71) nor were participant demographics (*F*_3,77_=14.74, Δ *R*^2^=.36, *P*<.001).

## Discussion

### Principal Results

The results of this study indicate that among individuals with no prior experience with mindfulness meditation, participant reports of their intentions to practice mindfulness meditation and subjective norms were the best predictors of the number of days and minutes spent practicing mindfulness using a phone app over a 2-week period. These results are generally consistent with the Theory of Planned Behavior (hypothesis 1), although subjective norms appeared to affect the number of minutes practicing mindfulness directly and not through intentions. Participant attitudes and perceived behavioral control were not associated with app usage in this study. Contrary to Temporal Self-Regulation Theory and our second hypothesis, executive functioning was not a predictor of app usage or a moderator of the intention-to-behavior link.

These results have implications that are particularly relevant for public health and medical practice. Among populations with limited experience with mindfulness meditation, creating more favorable subjective norms may be an important intervention focus to increase the amount of time that individuals spend practicing mindfulness (eg, number of minutes spent practicing mindfulness). This may be particularly true in younger populations where rational considerations such as knowledge and attitudes may play less of a role than affective considerations such as subjective norms [23]. Although subjective norms predicted practicing mindfulness in this study, the subjective norm-intention relationship was nonsignificant, which is consistent with other studies that have found that the link is not always strong [28]. For example, prior research has demonstrated a weaker association between subjective norms and intentions when measuring physical activity compared to studies measuring sexual and reproductive health behaviors [22].

Practitioners may be most successful at increasing intentions to practice mindfulness by targeting participant attitudes and perceived behavioral control. In this study, participant attitudes and perceived behavioral control were both positively associated with intentions, which led to increased consistency and time practicing mindfulness. Participants in our sample overwhelmingly held positive attitudes and reported high perceived behavioral control about mindfulness meditation. Thus, a little time spent in an intervention showing participants how to access mindfulness meditation apps and educating them on the benefits of mindfulness may go a long way in helping to increase their intentions to engage in mindfulness meditation.

Prior studies have demonstrated that executive functioning deficits have been associated with decreased adherence to treatment plans [36]. In this study, however, executive functioning performance (including working memory, inhibitory control, and cognitive shifting) explained almost no variance in the models and did not appear to interfere either positively or negatively with mindfulness meditation using a phone app. This finding may be particularly relevant for practitioners who use mindfulness meditation as a treatment or prevention option in their work in populations with suspected or known executive functioning deficits, such as among populations with mental health disorders or in communities with high stress and poverty [37-39]. Thus, an intriguing consideration is that phone apps might be an effective strategy to circumventing some of the challenges to adherence in populations with suspected executive functioning deficits.

Although the initial results are encouraging that executive functioning deficits may not undermine participant intention to practice mindfulness meditation, more research is needed. Examining the maintenance of mindfulness meditation practice over a longer period of time would be an important next research step because the mindfulness meditation practice in this study was only studied over 2 weeks. Furthermore, more randomized controlled trials to assess the effectiveness of mindfulness meditation phone apps are needed to examine their efficacy given the paucity of studies on this topic.

### Study Limitations and Strengths

This study was based on a convenience sample of undergraduate students attending a university with high admissions standards. Although a small subset of the sample scored below one standard deviation of the age-standardized mean for executive functioning ability, this was still a relatively high functioning, homogenous sample. As such, these results may not be generalizable to other young adult populations. Future studies using a more diverse, random sample would be beneficial. A second limitation of the study was the length between time points, which was only 2 weeks. It is possible that results may vary over time as the experience of practicing mindfulness becomes less novel. Thus, the study should be replicated over several weeks or months to see if executive functioning skills would play a bigger role.

Although there are limitations to the diversity of the sample, these results do provide novel data that future studies can build on. Specific strengths of the study included that we had a longitudinal design and used task measures of executive functioning. Further, the study provides one method for a way to collect engagement data from participants (eg, the use of a mindfulness log). Future studies may also investigate other methods such as built-in app usage data. Finally, this is the first study, to our knowledge, that examined theoretical predictors of mindfulness meditation using mobile phone apps. As such, the results may be particularly relevant to those developing health promotion and psychotherapy interventions using phone apps.

### Conclusions

The Theory of Planned Behavior appears to be a good theoretical framework for predicting mindfulness meditation app usage among participants with no prior experience with mindfulness. In particular, intentions to practice mindfulness and subjective norms directly predict both the number of days and number of minutes practicing mindfulness using a phone app. Participant attitudes and perceived behavioral control were associated with participant intentions to practice mindfulness. Participant executive functioning does not appear to influence mindfulness meditation app usage, either directly or as a moderator, suggesting that participant limitations in cognitive control capacity might not undermine mindfulness meditation interventions using phone apps.

## Abbreviations

CFI: comparative fit index
NIH-TB: NIH Toolbox
RMSEA: root mean square error of approximation
